# High-throughput microgel biofabrication via air-assisted co-axial jetting for cell encapsulation, 3D bioprinting, and scaffolding applications

**DOI:** 10.1088/1758-5090/acc4eb

**Published:** 2023-04-04

**Authors:** Vaibhav Pal, Yogendra Pratap Singh, Deepak Gupta, Mecit Altan Alioglu, Momoka Nagamine, Myoung Hwan Kim, Ibrahim T Ozbolat

**Affiliations:** 1 Department of Chemistry, Penn State University, University Park, PA 16802, United States of America; 2 The Huck Institutes of the Life Sciences, Penn State University, University Park, PA 16802, United States of America; 3 Engineering Science and Mechanics Department, Penn State University, University Park, PA 16802, United States of America; 4 Department of Biomedical Engineering, Penn State University, University Park, PA 16802, United States of America; 5 Materials Research Institute, Penn State University, University Park, PA 16802, United States of America; 6 Department of Neurosurgery, Penn State University, Hershey, PA 17033, United States of America; 7 Penn State Cancer Institute, Penn State University, Hershey, PA 17033, United States of America; 8 Department of Medical Oncology, Cukurova University, Adana 01130, Turkey; 9 Biotechnology Research and Application Center, Cukurova University, Adana 01130, Turkey

**Keywords:** microgels, coaxial nozzle, cell encapsulation, 3D bioprinting, scaffolding

## Abstract

Microgels have recently received widespread attention for their applications in a wide array of domains such as tissue engineering, regenerative medicine, and cell and tissue transplantation because of their properties like injectability, modularity, porosity, and the ability to be customized in terms of size, form, and mechanical properties. However, it is still challenging to mass (high-throughput) produce microgels with diverse sizes and tunable properties. Herein, we utilized an air-assisted co-axial device (ACAD) for continuous production of microgels in a high-throughput manner. To test its robustness, microgels of multiple hydrogels and their combination, including alginate (Alg), gelatin methacrylate (GelMA) and Alg–GelMA, were formed at a maximum production rate of ∼65 000 microgels s^−1^ while retaining circularity and a size range of 50–500 *µ*m based on varying air pressure levels. The ACAD platform allowed single and multiple cell encapsulation with 74 ± 6% efficiency. These microgels illustrated appealing rheological properties such as yield stress, viscosity, and shear modulus for bioprinting applications. Specifically, Alg microgels have the potential to be used as a sacrificial support bath while GelMA microgels have potential for direct extrusion both on their own or when loaded in a bulk GelMA hydrogel. Generated microgels showed high cell viability (>90%) and proliferation of MDA-MB-231 and human dermal fibroblasts over seven days in both encapsulation and scaffolding applications, particularly for GelMA microgels. The developed strategy provides a facile and rapid approach without any complex or expensive consumables and accessories for scalable high-throughput microgel production for cell therapy, tissue regeneration and 3D bioprinting applications.

## 1. Introduction

Recent advances in tissue engineering have shown microgels to be a progressively used versatile class of materials, as scaffolds or drug/bioactive molecule delivery platforms [1]. Microgels are a colloidal dispersion or suspension of gel-like particles consisting of a crosslinked network of polymer molecules. As opposed to a bulk gel, which is defined on the macroscopic scale as a state of matter, microgels are expressed on a microscopic scale (ranging from tens of nanometers to micrometers) and possess both polymeric and particle-like properties with high surface-to-volume ratio and rate of response to stimuli [2, 3]. They offer unique properties of injectability, porosity, interparticle interactions, highly adaptable modular microstructure, along with biocompatibility and drug and cell encapsulation capabilities. Their extensive tunability has made them applicable in a wide range of tissue engineering applications [4, 5]. Further, their performance for specific applications can be tuned by altering their physicochemical properties. Particularly, the size of microgels has a significant impact on their kinetic and thermodynamic properties as well as on their interactions with the surroundings [6]. Microgel size also directly influences the amount of drugs or cells that can be encapsulated inside and later released. Therefore, high-throughput biofabrication of microgels with uniform size and shape is urgently needed to meet criteria for various tissue engineering, biofabrication and therapeutic applications.

With increased interest in microgels, various microgel fabrication techniques have been developed and advanced including micromolding, emulsion-based, shearing, and extrusion techniques [1, 7, 8]. The use of these techniques depends on the final application and compatibility of the biofabrication process with the hydrogel’s gelation mechanism. Recently, the microfluidic emulsion technique is particularly prevalent because of its capacity for microscale fluid manipulation [9, 10]. This technique involves mixing two immiscible fluids—oil and a hydrogel precursor—to create droplets of the hydrogel precursor in oil using a flow-focusing device, typically a chip [11]. The ability to control the size and shape of the microgels makes microfluidics the method of choice for many applications. However, these systems suffer from high manufacturing costs coupled with an escalating level of system design complexity, limited microgel production rate, and the lack of universality for including various solvents [12]. Therefore, a method for producing monodisperse microgels in a high-throughput, cost-effective and rapid manner is still desired. There have been numerous attempts to create microscale particles made of polyelectrolyte complexes. Herrero *et al* developed a microencapsulation technology to produce sodium alginate particles (1–50 *µ*m) using compressed air [13]. Wang *et al* developed a miniature gas–liquid coaxial flow device using glass capillaries, aiming to produce sub-100 *μ*m calcium-alginate microspheres [14]. These are emerging as versatile tools for aiding in the regeneration of damaged tissues.

Recently, much research has been focused on using microgels as a bioink for 3D bioprinting applications by improving structural integrity and maintaining biological functions. This requires smooth extrusion of microgels as well as adequate mechanical strength to withhold 3D bioprinted constructs whilst preserving the viability of encapsulated cells [4, 5, 15]. In one of the initial reports on microgel fabrication, Du *et al* developed a bottom-up approach to control the assembly of cell-laden microgels to produce tissue constructs with adjustable complexity and microarchitecture. They used the thermodynamic tendency of multiphase liquid–liquid systems to minimize their contact surfaces to form poly(ethylene glycol) microgels [16]. Recently, alginate microgels have been used as a yield-stress support bath for aspiration-assisted bioprinting, which enabled the assembly of human mesenchymal stem cell spheroids for bone and cartilage tissue fabrication [17, 18]. Concurrently, using a flow-focusing microfluidic device, core–shell gelatin methacrylate (GelMA) microgels have been produced [19]. The results showed biocompatibility of microgels by encapsulating liver cells in the cores, with the ability to coculture liver cells and vascular endothelial cells, indicating that core–shell architectures are suitable for heterocellular cultures. Utilizing the injectable shear thinning capabilities of microgels can help create highly customizable systems that can be used for cell and tissue (i.e. islet) delivery [20, 21]. 3D bioprinted microgels are emerging as promising platforms for generating tissue substitutes. To control the printability of the components and modulate desired physicochemical characteristics of the constructs more efficiently, development of bioinks based on jammed particles has been demonstrated [22]. Recently, Ouyang *et al* developed a microgel-templated porogel bioink platform for controlled microporosity. Thermosensitive gelatin microgels were fabricated and mixed with different photo-crosslinkable formulations, where eventually gelatin would dissolve and diffuse out at the physiological temperature forming micropores. In addition, gelatin microgels significantly enhanced the printability of bioinks at various cell densities [23]. The approach enables the engineering of highly tunable pores in cell-laden bioinks with controlled porosity and pore size; however, the fabrication process is conventional and low-throughput, and the removal of gelatin microgels by diffusion is a slow process.

**Figure 1.**
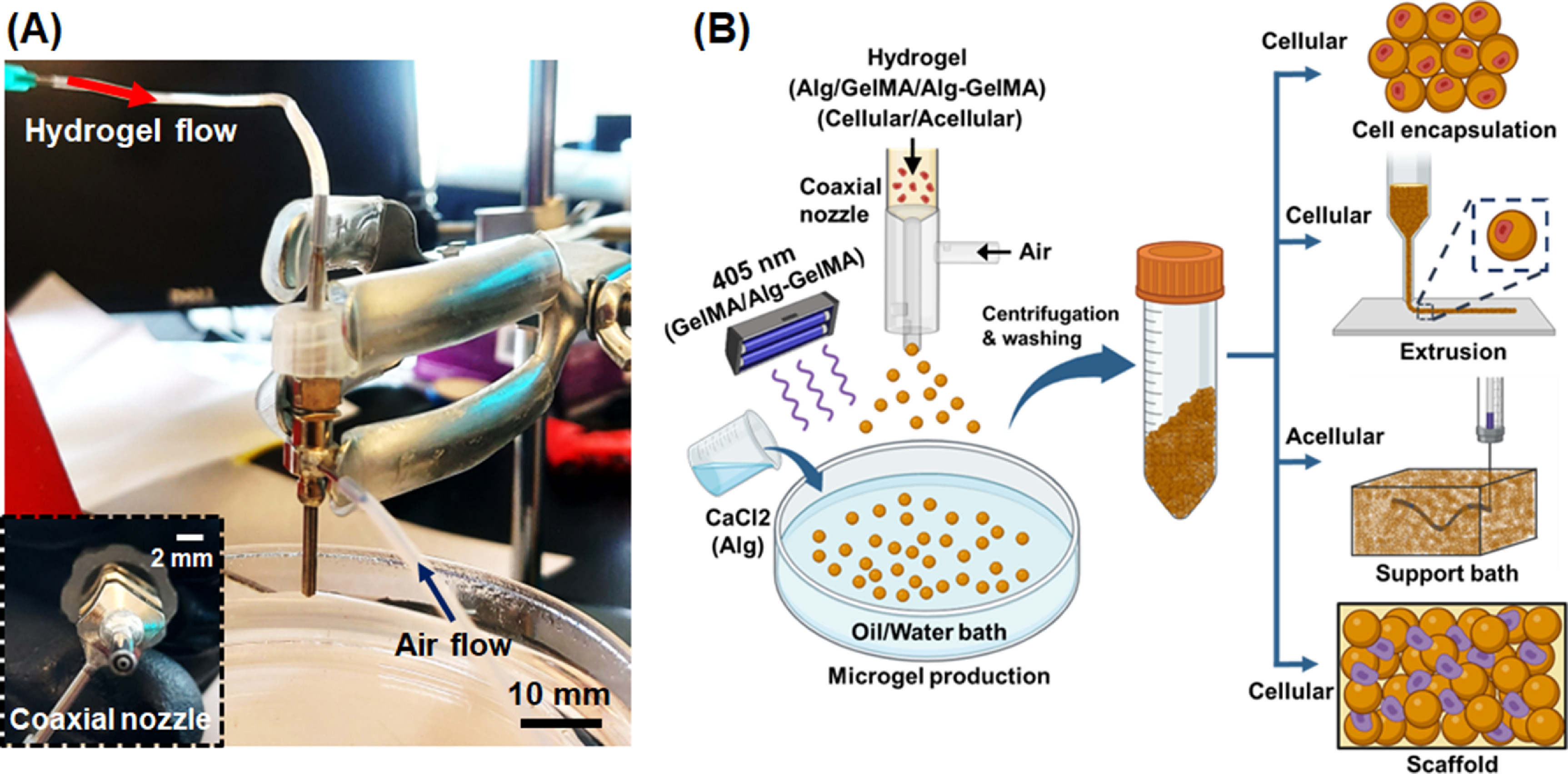
High-throughput fabrication of microgels. (A) The experimental setup, which involves a coaxial nozzle that was attached to an air pump for air flow and a syringe pump for hydrogel flow, where a collector bath was maintained below the coaxial nozzle (scale bar with respect to the coaxial nozzle). (B) The schematic diagram of microgel production from Alg, GelMA and Alg–GelMA hydrogels using the ACAD device. Microgels were collected, washed, and centrifuged for multiple applications including cell encapsulation, bioprinting via direct extrusion of cell encapsulated microgels or support bath for embedded bioprinting, and scaffolding.

Herein, we utilized an air-assisted co-axial device (ACAD) to produce cell-laden or acellular microgels. The device setup had a concentric arrangement of two separate nozzles through which the polymer solution and air flowed (figure 1). To demonstrate the versatility of the process, microgels of multiple materials and their combination were fabricated including alginate (Alg), GelMA (GelMA), and alginate–GelMA (Alg–GelMA) microgels. The formed microgels were tested for multiple applications including their utilization for cell encapsulation, bioprinting (as a bioink for direct extrusion or as a support bath for embedded bioprinting), and scaffolding.

## 2. Materials and methods

### 2.1. Device fabrication and setup

The developed ACAD platform was purpose-built for microgel production and had four main components as shown in figure 1(A). The first component was the coaxial nozzle system, where one nozzle was used for material flow and the other one for airflow. The second component was the air controller system (Ultimus I, Nordson EFD, East Providence, RI), which was used to regulate airflow. The air controller system was connected to the outer nozzle (15 G) of the coaxial assembly. The third component was a syringe pump (New Era Pump System Inc., Farmingdale, NY) that was used to drive the syringe plunger to push the hydrogel through a tube, which was connected to the inner nozzle (21 G) of the coaxial assembly. The last component was the collection bath, which was filled with oil or calcium chloride (CaCl_2_) solution as per the precursor hydrogel used. The collection bath was utilized for the collection and crosslinking of microgels. Along with these main components, a magnetic stirrer was used to homogeneously stir the solution in the collection bath. The collection bath was placed on the magnetic stirrer below the coaxial nozzle system, which was fixed with a clamp. Once the setup was built, precursor hydrogel solutions were prepared and microgels were generated and then crosslinked as shown in figures 1(B), S1 and video S1. The air pressure and flow rate were regulated and optimized to produce different types and sizes of microgels.

### 2.2. Preparation of alginate (Alg) microgels

Alginate microgels were produced by dissolving sodium alginate (Sigma-Aldrich, United Kingdom (UK)) in deionized (DI) water at different concentrations (0.5%, 1%, and 2% w/v). CaCl_2_ solution was prepared by dissolving 4% (w/v) CaCl_2_ (Sigma-Aldrich, St. Louis, MO) in DI water. The alginate solution was taken into a 10 ml syringe and extruded through the inner part of the coaxial nozzle at flow rates of 10, 50, 100, and 500 *µ*l min^−1^, and air pressure levels of 40, 60, 100, and 180 kPa. After optimization, the flow rate was finalized at 100 *µ*l min^−1^ and the air pressure was varied as mentioned before to regulate the size of microgels. The generated microgels were collected in the CaCl_2_ bath for crosslinking. After generating microgels, they were transferred into a 50 ml centrifuge tube and washed thrice using DI water to remove the excess CaCl_2_ and uncrosslinked alginate. The collected microgels were used for further studies.

### 2.3. Preparation of GelMA microgels

GelMA was synthesized as per previously reported protocol [24], by reacting methacrylic anhydride with gelatin, purified using 12–14 kDa cut-off dialysis membrane, and freeze-dried. Both fish GelMA and porcine GelMA were synthesized from gelatin from cold fish water skin (Sigma-Aldrich) and type A gelatin from porcine skin (Sigma-Aldrich), respectively. The freeze-dried GelMA was reconstituted to a 10% (w/v) final solution. The 10% GelMA solution from both fish and porcine sources was mixed at a 2:8 ratio, respectively, and then 0.5% lithium phenyl (2,4,6-trimethylbenzoyl) phosphinate (LAP) (TCI chemicals, OR) was added. The formed GelMA solution was loaded into a 10 ml syringe and extruded at an air pressure of 40, 60, 100, and 180 kPa. Light mineral oil (Sigma-Aldrich, St. Louis, MO) with nonionic surfactant (3% Span 80, Sigma-Aldrich) was used in the collection bath to collect GelMA microgels. A visible or near-ultraviolet (UV) light source (GHDO, SOVOL, Shenzhen, China) of 405 nm was used to crosslink GelMA droplets to form microgels. Only the oil/water collection bath was exposed to the light source during microgel production. The formed microgels were washed three times with DI water followed by centrifugation to remove the oil and surfactant. The collected microgels were used for further studies.

### 2.4. Preparation of Alg–GelMA microgels

Alg–GelMa microgels were produced using sodium alginate, fish GelMA, and porcine GelMA in DI water. A GelMA solution consisting of 10% Fish GelMA:10% Porcine GelMA (2:8) was taken and mixed with 1% alginate solution in a 1:1 ratio. The resulting solution was then used for microgel fabrication. Air pressure levels and flow rates were maintained the same as with the above-mentioned microgels. Since the solution consisted of both GelMA and alginate, photocrosslinking (using 405 nm light) and ionic crosslinking (using CaCl_2_ solution) were used simultaneously to crosslink GelMA and alginate, respectively. These microgels were transferred into a 50 ml centrifuge tube and washed thrice using DI water to remove the excess CaCl_2_ and uncrosslinked Alg–GelMA. The collected microgels were used for further studies.

### 2.5. Rate, shape, size, topography, and stability characterization

To quantify the rate of microgel production, microgels were produced for 2 min and collected in 20 ml of the respective collection bath. The number of microgels was counted using a hemocytometer (ART. No. 1280, Ningbo Hinotek Technology Co., China). To assess the morphology of microgels, circularity was calculated using ImageJ software (National Institute of Health, Bethesda, MD). The value ‘0’ indicated an infinitely elongated polygonal shape and ‘1’ indicated a perfectly circular shape.

Further, laser granulometry with a Mastersizer 3000 (Malvern Panalyticals, Worcester, UK) was carried out to assess the size of microgels, which was quantified as volume-weighted mean particle size. The particle size analysis was done for samples produced at a constant flow rate of 100 *µ*l min^−1^ and different polymer concentrations and air pressure levels as mentioned before to study the dependence of particle size distribution on the air pressure.

The surface topography of prepared microgels was assessed using field emission scanning electron microscopy (Apreo S, Thermo Fisher Scientific). The samples were dehydrated using graded ethanol solutions, 20%, 50%, 60%, 70%, 80%, 90%, and 100%, sequentially each for 10 min. To ensure the complete removal of water, samples were further dried in a critical point dryer (CPD300, Leica, Wetzlar, Germany). The dehydrated samples were sputter-coated with iridium using a sputter coater (Leica, Wetzlar, Germany) and imaged at an accelerating voltage of 3–5 kV.

The stability of generated microgels was evaluated qualitatively by assessing their degradation for 21 days. For this, Alg, Alg–GelMA, and GelMA microgels were generated, and 250 *µ*l of each microgel was mixed with 2 ml PBS, and the resulting mixture was transferred to well plates and incubated at 37 °C. Optical images were taken using a Zeiss Axio Observer microscope (Zeiss, Jena, Germany) on Days 1, 3, 5, 7, 14, and 21.

### 2.6. Rheological analysis

Rheological properties of microgels were characterized with a rheometer (MCR 302, Anton Paar, Austria). Triplicates were taken for each sample. The tests were carried out using a 25 mm parallel-plate geometry measuring system maintained at 22 °C with a Peltier temperature control system. The shear thinning behavior of Alg, GelMA, Alg–GelMA microgels was evaluated by performing a flow sweep test, where the shear rate was varied from 0.1 to 100 s^−1^. To evaluate the viscoelasticity of microgels, amplitude sweep tests were performed at a constant frequency of 1 Hz and shear strain was varied from 0.1% to 100%. The frequency sweep tests were executed to study the storage modulus (*G*′) and loss modulus (*G*″) within the linear viscoelastic region at a low shear strain of 0.1% to prevent damage to samples and the angular frequency was varied from 0.1 to 100 rad s^−1^. Further, self-healing tests were performed by measuring the viscosity of microgels under alternating low and high shear rates of 0.1 s^−1^ for 60 s and 100 s^−1^ for 10 s, respectively.

### 2.7. Generation of cell-encapsulated microgels

To generate cell-encapsulated microgels, the ACAD platform was prepared under sterile conditions where a 0.22 *µ*m filter was used to sterilize the air and precursor polymer solutions, which were then loaded with green fluorescent protein (GFP^+^) MDA-MD-231 breast cancer cells and jetted using the ACAD platform. Additionally, the lyophilized or powdered polymers were UV sterilized and then dissolved under sterile conditions. The whole setup including the 3D bioprinter, pumps, tubing, water/oil bath was maintained under a Biosafety Level-2 (LabGard^®^ ES (Energy Saver) Class II, Type A2 Plymouth, MN) safety cabinet. The hydrogel flow rate was maintained constant at 100 *µ*l min^−1^ and the air pressure was maintained at 100 kPa for Alg and GelMA, and 60 kPa for Alg–GelMA. The reduced air pressure for Alg–GelMA was a result of their inability to form stable microgels at higher pressure levels. For cell encapsulation in microgels, GFP^+^ MDA-MB-231 cells (5 × 10^6^ cells ml^−1^) were pre-mixed in the precursor polymer suspensions and formed into microgels. Once cell-laden microgels were generated and crosslinked, they were washed with DI water three times and centrifuged at 2000 rpm and dispersed into cell media. They were cultured in Dulbecco’s Modified Eagle Medium media supplemented with 10% fetal bovine serum (Life Technologies, Grand Island, NY), 1 mM Glutamine (Life Technologies, Carlsbad, CA) and 1% Penicillin–Streptomycin (Life Technologies). MDA-MB-231 cells were used at passages 56 through 64. The formed cell-encapsulating microgels were imaged using the Axio Observer microscope after 6 h of culture.

The encapsulation efficiency was assessed using the images, where the total number of cell-encapsulating microgels was divided by the total number of microgels to give the percent efficiency. For each sample, three different sites were imaged from three independent experiments resulting in a total of nine images, and the encapsulation was confirmed by the presence of green fluorescence emitted by GFP^+^ cells inside each microgel. Microgel containing one or more GFP^+^ cells were considered positive for encapsulation. Further, to assess the average number of cells per microgel, a similar method was followed and the number of GFP^+^ cells per microgel was counted and the ratio was reported accordingly.

In addition, biocompatibility of microgels was also demonstrated using human dermal fibroblasts (HDFs). Herein, both HDFs and MDA-MB-231 were separately loaded into GelMA microgels, which were then later loaded in bulk GelMA. To assess the viability and proliferation of these cells, an AlamarBlue dye reduction assay (Invitrogen, USA), which was indicative of metabolically active cells, was performed following the manufacturer’s protocol. Briefly, samples were incubated with 10% (v/v) of the dye for 3 h. After incubation, 100 *μ*l of the culture media was read using a microplate reader (Tecan Infinite 200 Pro, Switzerland) at 570/600 nm (excitation/emission). The results were presented as the normalized value of the dye reduced, which was proportional to the number of viable cells present in microgels.

Further, to assess the feasibility of using different cell types, HDF-loaded GelMA microgels were bioprinted into 6 × 6 mm double-layer grids. To assess cellular viability, bioprinted constructs were treated with a Calcein-AM (2 *μ*M)/ethidium homodimer-1 (EthD-1, 4 *μ*M) LIVE/DEAD assay kit (Invitrogen, CA) post 24 h of culture. The stained cells were observed using the Axio Observer microscope (Zeiss, Jena, Germany). Six different samples (*n* = 6) were imaged and the viability was quantified using ImageJ.

### 2.8. Extrudability and shape fidelity analysis

#### 2.8.1. Hanging filament test

Fabricated GelMA microgels were added into 15% porcine GelMA hydrogel in a 1:1 ratio, and then mixed with a vortex mixer. Next, the suspension was centrifuged at 13 000 rpm and the supernatant was removed. This process was repeated twice and the centrifuged GelMA microgels in GelMA hydrogel were used for 3D bioprinting purposes.

Both GelMA microgels alone and GelMA microgels loaded in bulk GelMA were used as a bioink and tested for their maximum filament length to assess their extrusion fidelity for 3D bioprinting. The bioink made of GelMA microgels was filled to a 3 ml syringe barrel and extruded through a needle with the help of controlled pneumatic pressure using an Inkredible^+^ bioprinter (CELLINK, Sweden). The bioink came out in the form of filament and the maximum length of extruded filaments was measured before they broke. The needles used for this test were 25 G (250 *µ*m inner diameter), 22 G (410 *µ*m inner diameter), and 20 G (610 *µ*m inner diameter). The pneumatic pressure was maintained at 10–20 kPa for the bioink with GelMA microgels only, and 90–120 kPa for the GelMA microgels loaded in GelMA. The videos of filament extrusion were recorded using a Nikon D810 camera with Nikon 105 Micro lens. The frame, at which the filament broke its contact with the needle was taken and the length of the filament was measured using ImageJ.

#### 2.8.2. Filament fusion test

To perform the filament fusion test, grids with different pore sizes were 3D bioprinted and the spreading and printability analysis was performed according to the literature [25]. Briefly, a bi-layered grid with square pores of varying size from 1 × 1 to 5 × 5 (mm × mm) was bioprinted on glass slides. Both bioinks, i.e. GelMA microgels and GelMA microgels loaded in GelMA, were extruded through a 22 G needle at a bioprinting speed of 6 mm s^−1^. The former was extruded at a pressure of 15 kPa while the latter was extruded at 100 kPa. The images of bioprinted grids were taken with the Nikon D810 camera with Nikon 105 Micro lens and analyzed using ImageJ. The material spreading (*M_s_*) was calculated using equation (1), which allows one to interpret the degree of bioink spreading after extrusion. The percentage diffusion was ‘0’ in case the bioink did not spread and the actual area became equal to the theoretical area. Here *S*_t_ and *S*_a_ were the theoretical and actual area of the pores, respectively. Further, the printability of the bioinks was calculated using equation (2), where ‘1’ indicated perfect printability

\begin{equation*}{M_s} = \left( {{S_{\text{t}}} - {S_{\text{a}}}} \right)/{S_{\text{t}}} \times 100\% \end{equation*}

\begin{equation*}Pr = {L^2}/\left( {16{S_{\text{a}}}} \right).\end{equation*}

In the equation, *L* denoted the perimeter of the actual pore.

#### 2.8.3. Filament collapse test

The filament collapse test was performed by bioprinting filaments over a linear array of pillars at distances of 1, 2, 3, 4, 5, and 6 mm [25]. Bioprinted filaments were photographed and the collapse area factor (*C_f_*, the percentage of deflected area compared to the theoretical area) was determined using equation (3):

\begin{equation*}{C_f} = \frac{{{A_{\text{t}}} - {A_{\text{a}}}}}{{{A_{\text{t}}}}} \times 100\end{equation*}

where, *A*_a_ and *A*_t_ were actual and theoretical areas, respectively. In the case of collapsed filaments, *A*_a_ was taken to be zero, thus *A*_t_ was 100%.

#### 2.8.4. Shape fidelity analysis

The shape fidelity test was performed on 3D printed hollow cylindrical structures with a diameter of 5 mm and a height of 5 or 10 mm [26]. Depending on the filament size and cylinder height, 15 or 30 layers were deposited using a 22 G nozzle. The images of the 3D printed structures were captured by a Nikon D810 camera, and the analysis was performed using ImageJ. The height maintenance factor (*H_m_*) was measured as the ratio of actual height (*H*_a_) to the theoretical height (*H*_t_) using equation (4)

\begin{equation*}{H_m} = {H_{\text{a}}}/{H_{\text{t}}}.\end{equation*}

### 2.9. 3D bioprinting

GFP^+^ MDA-MB-231 cell-loaded GelMA microgels were 3D bioprinted on a build-plate at room temperature using the Inkredible^+^ bioprinter with a 22 G needle. The bioprinting speed and pneumatic pressure were maintained at 6 mm s^−1^ and 15 kPa, respectively.

In addition, Alg microgels were explored as a support bath for embedded bioprinting to form complex branched structures. In this regard, Alg microgels were cast into a transparent container. To demonstrate the support bath capabilities of Alg microgels, we used embedded bioprinting. For this, xanthan gum was chosen as the bioink material due to its shear thinning properties [27]. A 2% w/v xanthan gum (from *Xanthomonas campestris*, Sigma Aldrich) was prepared in a commercial blender (Magic bullet, Homeland Housewares, CA) for 3 min followed by centrifugation at 4000 rpm for 5 min. Since the xanthan gum bioink was transparent, oil-based red dye with surfactant was added to make it visible during and after bioprinting. Alginate microgels were cast into a transparent container and the red-dyed xanthan gum was used to print a 3D structure inside the support bath with the Inkredible^+^ bioprinter. The needle used for embedded bioprinting was 25 G and the bioprinting speed was maintained at 1 mm s^−1^.

To assess the feasibility of 3D bioprinting of scalable complex-shaped constructs, a triple crown structure of 6 mm diameter and 6 mm height was bioprinted inside the Alg support bath using a 22 G nozzle and a bioprinting speed of 2 mm s^−1^ [28]. The bioink comprised GFP^+^ MDA-MB-231-encapsulated GelMA microgels loaded in bulk GelMA. The bioprinted constructs were photographed using the Nikon D810 camera and a Zeiss AxioZoom V16 Stereo fluorescent microscope (Zeiss, Jena, Germany) was used to visualize GFP^+^ MDA-MB-231 cells.

### 2.10. Microgels for scaffolding application

The formed acellular microgels were seeded with GFP^+^ MDA-MB-231 cells (10 × 10^6^ cells ml^−1^). The distribution of cells in the microgel matrix was assessed after 6 h using a Leica SP8 DIVE multiphoton microscope (Leica Microsystems, Germany) with a 16× water immersion lens. Dead cells were assessed by staining with ethidium homodimer-1 (EthD-1, 4 *µ*M) for 30 min in the incubator, which was then imaged using the Zeiss Axio Observer microscope. Images were analyzed using ImageJ to determine the red fluorescence intensity and the cellular viability was quantified by dividing the fluorescence intensity of dead cells (red) by the combined fluorescent intensity of both dead (red) and live (green) cells.

For evaluation of cell proliferation, MDA-MB-231 cells were seeded with microgels on cell-repellent surface microplates and assessed on Days 1, 3, and 7. The AlamarBlue dye reduction assay was used following the manufacturer’s protocol as discussed before in section 2.7 in order to assess the metabolic activity of cells.

### 2.11. Statistical analysis

All data were presented as mean ± standard deviation. Data were analyzed by OriginPro 9.1. Statistical differences were determined with one-way analysis of variance and Tukey’s post hoc test, and the analysis, if fulfilling the null hypothesis at *p* ⩽ 0.05, was considered as statistically significant (^*^), while at *p* ⩽ 0.01 (^**^) and *p* ⩽ 0.001 (^***^) as highly significant and ‘ns’ represents not significant.

## 3. Results

### 3.1. Fabrication of microgels and their physical characterization

**Figure 2.**
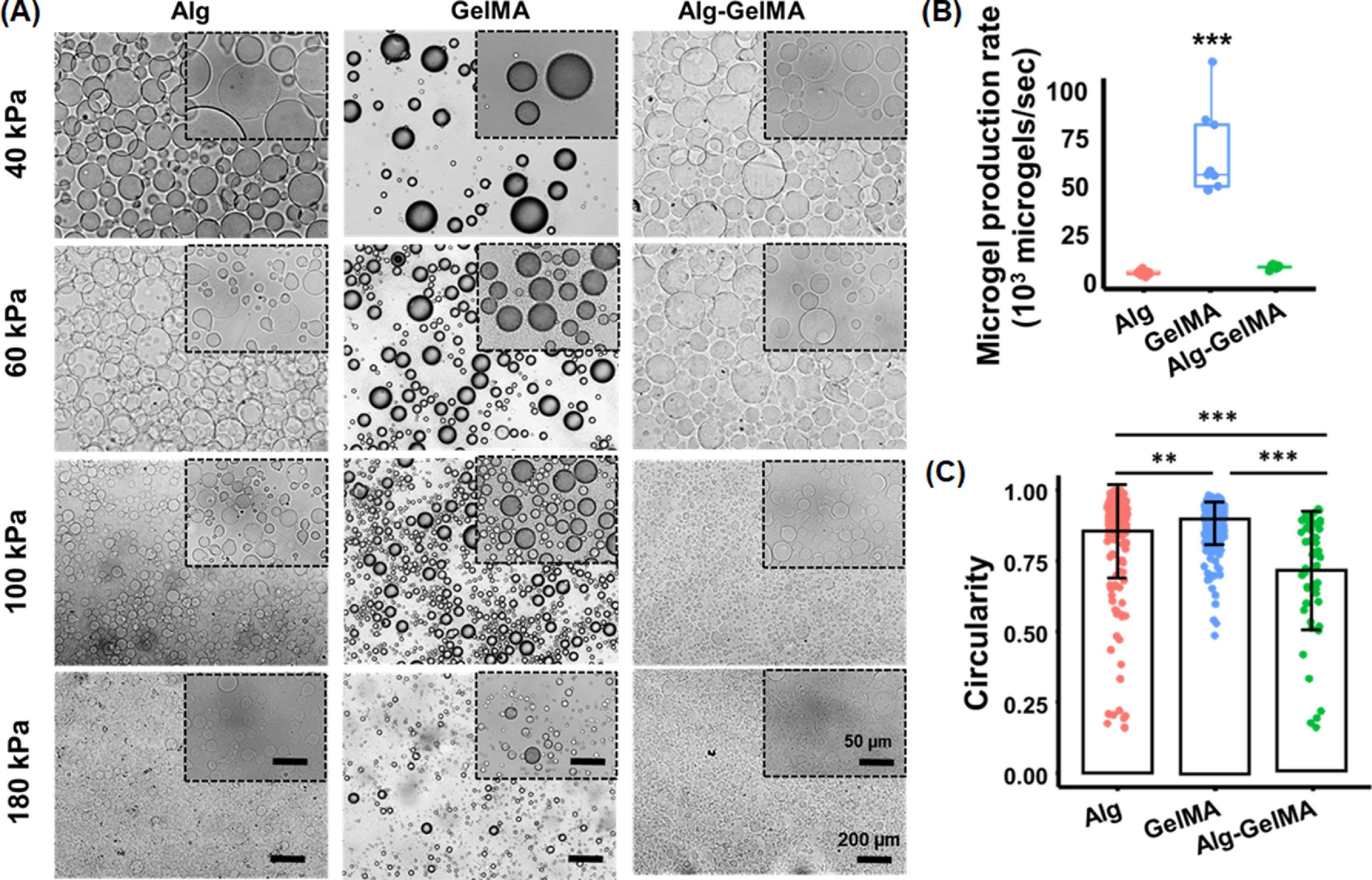
Morphological assessment and physical characterization of acellular microgels. (A) Microscopic images of Alg, GelMA, and Alg–GelMA microgels at different air pressure levels for visualization and comparison of shape and size (scale bar: 200 *µ*m); images in the inset show microgels at a higher magnification (scale bar: 50 *µ*m). (B) Microgel production rate (*n*= 9). (C) Quantitative assessment of circularity for the generated microgels (different number of microgels were considered for Alg (*n*= 222), GelMA (*n*= 252) and Alg–GelMA (*n*= 54) (*p* ^**^ < 0.01, *p* ^***^ < 0.001).

Spherical microgels of Alg, GelMA, and Alg–GelMA were successfully fabricated using the ACAD platform. The precursor polymer solutions were extruded at different pressure levels namely 40, 60, 100, and 180 kPa, and the microgel size and shape was analyzed. The brightfield images of Alg, GelMA, and Alg–GelMA microgels fabricated using above-mentioned pressure levels were shown in figure 2(A). The microgels exhibited spherical morphology and were separated from each other without any visible agglomeration. The efficacy of the ACAD platform was assessed by quantifying the rate of microgel production. The microgel production rate was found to be ∼3800 microgels s^−1^ for Alg, ∼65 000 microgels s^−1^ for GelMA, and ∼7000 microgels s^−1^for Alg–GelMA (figure 2(B)). The rate of production for GelMA microgels was significantly higher (*p* ⩽ 0.001) as compared to that for Alg and Alg–GelMA microgels. Further, the circularity of the microgels was determined (figure 2(C)). Alg, GelMA and Alg–GelMA microgels showed a circularity distribution ranging from 0.2 to 1, from 0.5 to 1 and from 0.2 to 1 respectively. However, depending on the range, the circularity of GelMA was determined to be significantly higher than that for Alg (*p* ⩽ 0.01) and Alg–GelMA (*p* ⩽ 0.001). Overall, the majority of Alg and GelMA microgels exhibited a circularity score of ∼1, indicating a perfect circular shape. Alg–GelMA microgels were large and less circular, which was also confirmed by SEM micrographs (figure S2).

**Figure 3.**
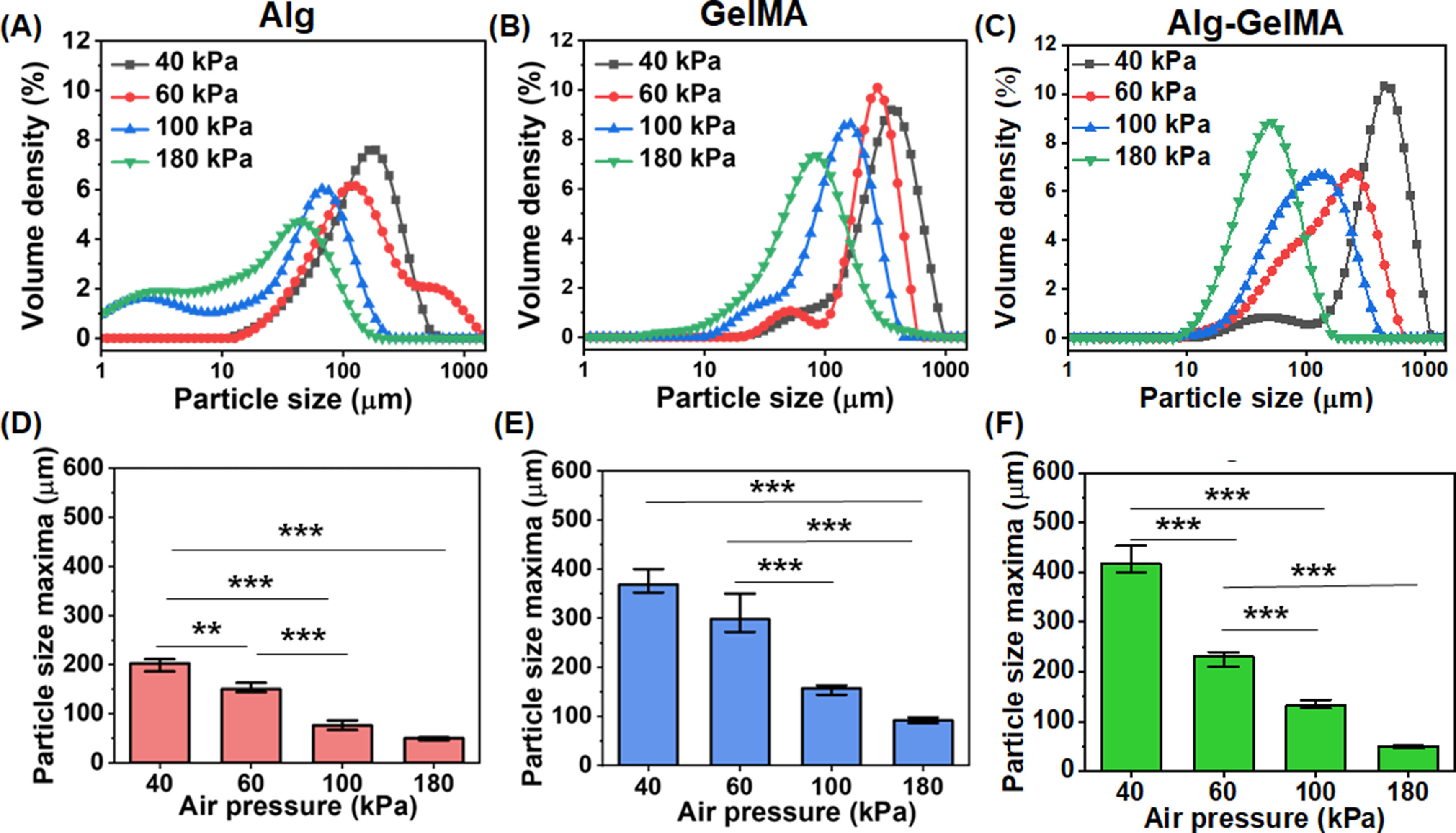
Particle size distribution analysis of microgels under different air pressure levels and a fixed flow rate. The volume density distribution profiles and particle size maxima of (A), (D) Alg, (B), (E) GelMA, (C), (F) Alg–GelMA microgels (*n*= 3; *p* ^**^ < 0.01, *p* ^***^ < 0.001).

The particle size distribution was assessed using a particle size analyzer and the plots were presented in figures 3(A)–(C). Different polymer concentrations were used to produce microgels and the size for these were measured (figure S3). Based on the peak maxima, the concentrations of 1% for Alg, 10% for GelMA, and a 1:1 mixture for Alg–GelMA blend were used for further studies. Microgels with sizes ranging from 10 to 1000 *μ*m were obtained based on varying air pressure levels. Table S1 shows the average size of these microgels. The microgel size decreased with increasing pressure levels. It was noted that the particle size maxima of the microgels decreased from 202 ± 14 to 49 ± 4 *μ*m for Alg, 368 ± 27 to 91 ± 6 *μ*m for GelMA, and 418 ± 31 to 49 ± 4 *μ*m for Alg–GelMA with an increase in pressure from 40 to 180 kPa (figures 3(D)–(F)). Alg microgels were relatively smaller in size followed by GelMA and Alg–GelMA counterparts at each pressure level. After generating all microgels at different pressure levels, we noticed the generation of larger microgels at lower pressure levels (40, 60 kPa), which were not suitable for bioprinting applications while smaller size microgels were observed at a high-pressure level (180 kPa) that might impede cell encapsulation. Therefore, we preferred 100 kPa air pressure for Alg and GelMA microgels. For Alg–GelMA microgels, we used 60 kPa since we did not get stable microgels at 100 kPa. Furthermore, the formed GelMA microgels were stable as indicated by their structure retention over 21 days (figure S4). On the other hand, Alg microgels were stable for up to 12 days while Alg–GelMA microgels were stable only for five days.

**Figure 4.**
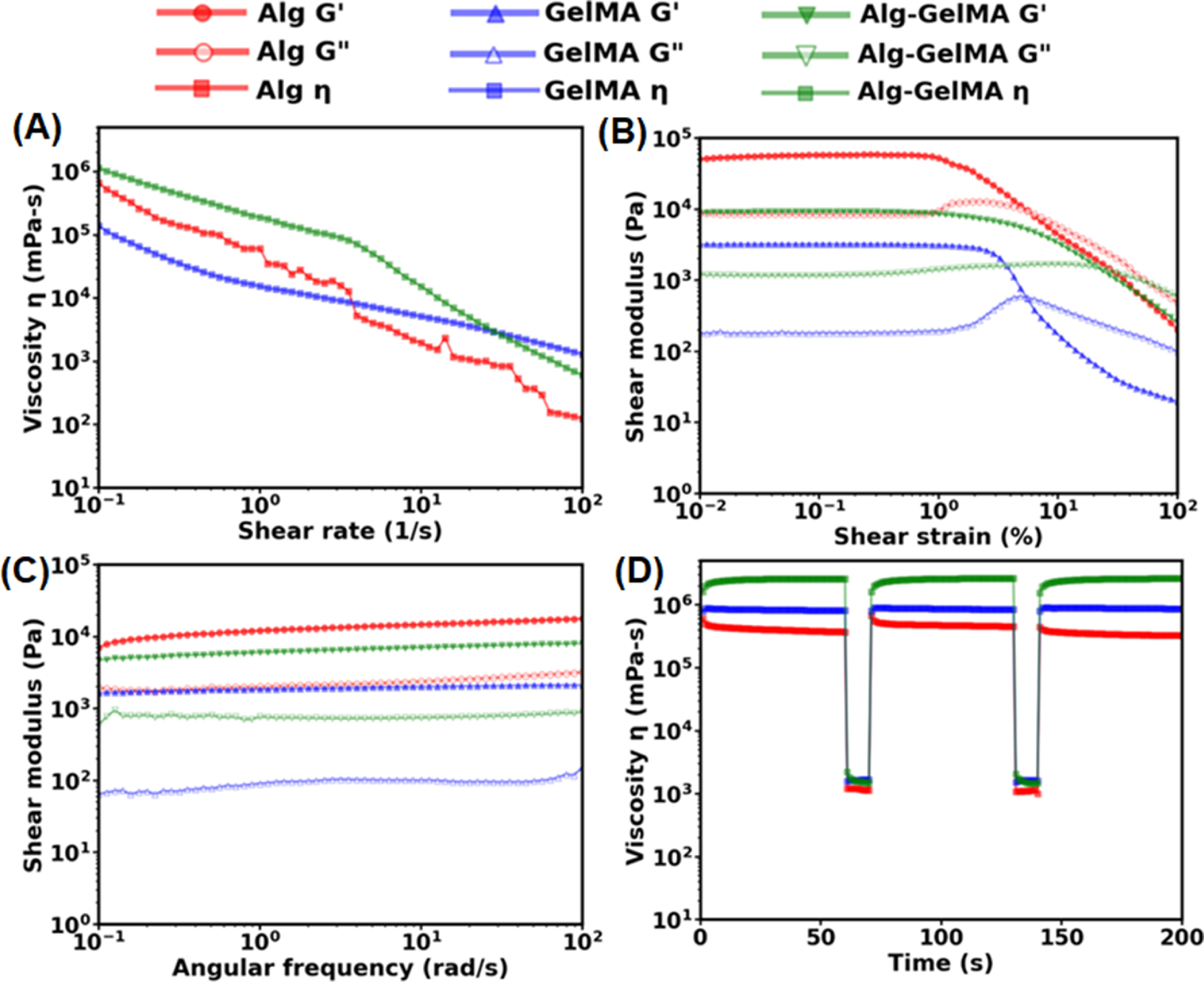
Rheological characterization of microgels. (A) Flow curves showing viscosity as a function of shear rate ranging from 0.1 to 100 s^−1^. (B) Amplitude sweep test to measure shear modulus of microgels at a strain ranging from 0.01 to 100%. (C) Frequency sweep test of microgels in the linear viscoelastic region at an angular frequency ranging from 0.1 to 100 rad s^−1^. (D) Self-healing tests to assess the recovery of microgels after applying a high shear stress. The viscosity was measured at five intervals at alternating shear rates of 0.1 s^−1^ for 60 s and 100 s^−1^ for 10 s.

Rheological assessment of microgels was performed to confirm that they were appropriate for specific applications such as an extrudable bioink or a support bath for 3D bioprinting. The shear thinning behavior of microgels was studied with a flow sweep test by measuring their viscosity at varying shear rates ranging from 0.1 to 100 s^−1^. All groups possessed shear thinning behavior and the viscosity decreased with increasing shear rate. The viscosity of Alg–GelMA was the highest up to a shear rate of 10 s^−1^ and decreased rapidly after that and became the lowest after 20 s^−1^ (figure 4(A)). The viscosities of Alg and GelMA microgels were nearly similar in the entire test range.

The amplitude sweep test demonstrated the transition of microgels from an elastic state (*G*′ > *G*″) to viscous state (*G*″ > *G*′) as strain was increased from 0.01% to 100%. All the samples initially showed higher *G*′ than *G*″ stating that microgels behaved as elastic materials under low values of shear strain and transitioned to a viscous state as the shear strain increased. It also illustrated that the storage modulus of Alg, GelMA, Alg–GelMA microgels was ∼250, ∼2, and ∼10 kPa, respectively, at low shear strain values until they reached their yield stress point, which is defined as the point where the microgels transitioned from elastic to viscous state (figure 4(B)). The percentage strain at which the material yields was found to be the highest for Alg–GelMA (20%), followed by GelMA (7%) and Alg (0.5%). The frequency sweep test demonstrated *G*′ as superior to *G*″ for all microgels indicating them as elastic materials at low shear strain of 0.01% and over the entire range of angular frequency 0.1–100 rad s^−1^ (figure 4(C)).

The self-healing test was also performed to assess the recovery of microgels after deformation that occurs because of a high shear rate particularly when using them as a support bath. The self-healing properties were studied by varying the shear rate from low (0.1 s^−1^) to high (100 s^−1^) alternatively in cycles. The low shear rate was applied to mimic the static condition while the high shear rate was applied to mimic the stress generated during the movement of a needle in a support bath. The healing behavior was observed by studying the difference in the viscosities before and after applying the high shear rate. As shown in figure 4(D), it was found that all microgel types recovered completely, indicating their self-healing behavior. The change in the viscosity was more substantial for Alg–GelMA microgels compared to their Alg and GelMA counterparts.

### 3.2. Applications of Alg, GelMA and Alg–GelMA microgels

To exemplify the utility of fabricated microgels, we demonstrated three distinct applications including cell encapsulation, bioprinting (both direct extrusion of microgels and their use as a support bath) and scaffolding.

**Figure 5.**
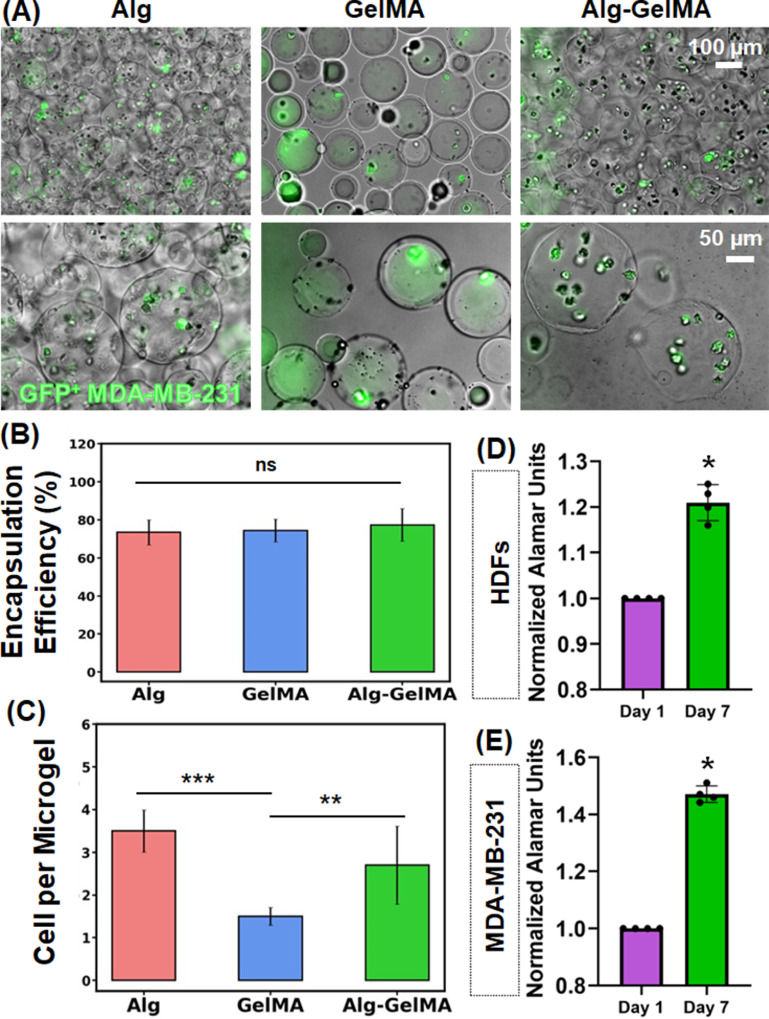
Cell encapsulation. (A) Fluorescence microscopy images showing GFP^+^ MDA-MB-231 cells encapsulated in microgels. (B) Cell encapsulation efficiency and (C) the number of cells per microgel in Alg, GelMA, and Alg–GelMA microgels (*n*= 5). (D) AlamarBlue reduction (normalized to Day 1) indicating the proliferation and metabolic activity of HDF and MDA-MB-231 cell-encapsulated GelMA microgels loaded in bulk GelMA over a week (*n*= 4; *p* ^*^ < 0.05, *p* ^**^ < 0.01, *p* ^***^ < 0.001, ns—not significant).

In the first application, we demonstrated cell encapsulation. Upon combining GFP^+^ MDA-MB-231 cells with the precursor polymer solution, cell-encapsulated microgels were produced as shown in figure 5(A), S5. The encapsulation efficiency of MDA-MB-231 cells in microgels was found to be ∼75% for all three microgel types (figure 5(B)). Further, the number of cells per microgel ratio was ∼3.2 for Alg, ∼1.5 for GelMA, and ∼2.7 for Alg–GelMA microgels (figure 5(C)). In addition, the biocompatibility of microgels and the feasibility of the presented strategy was also confirmed using another cell type, HDFs. Cell (HDF or MDA-MB-231) encapsulated GelMA microgels loaded in bulk GelMA supported the growth of HDFs and MDA-MB-231 cells, where both cell types considerably proliferated at Day 7 as compared to Day 1 (figures 5(D) and (E)).

**Figure 6.**
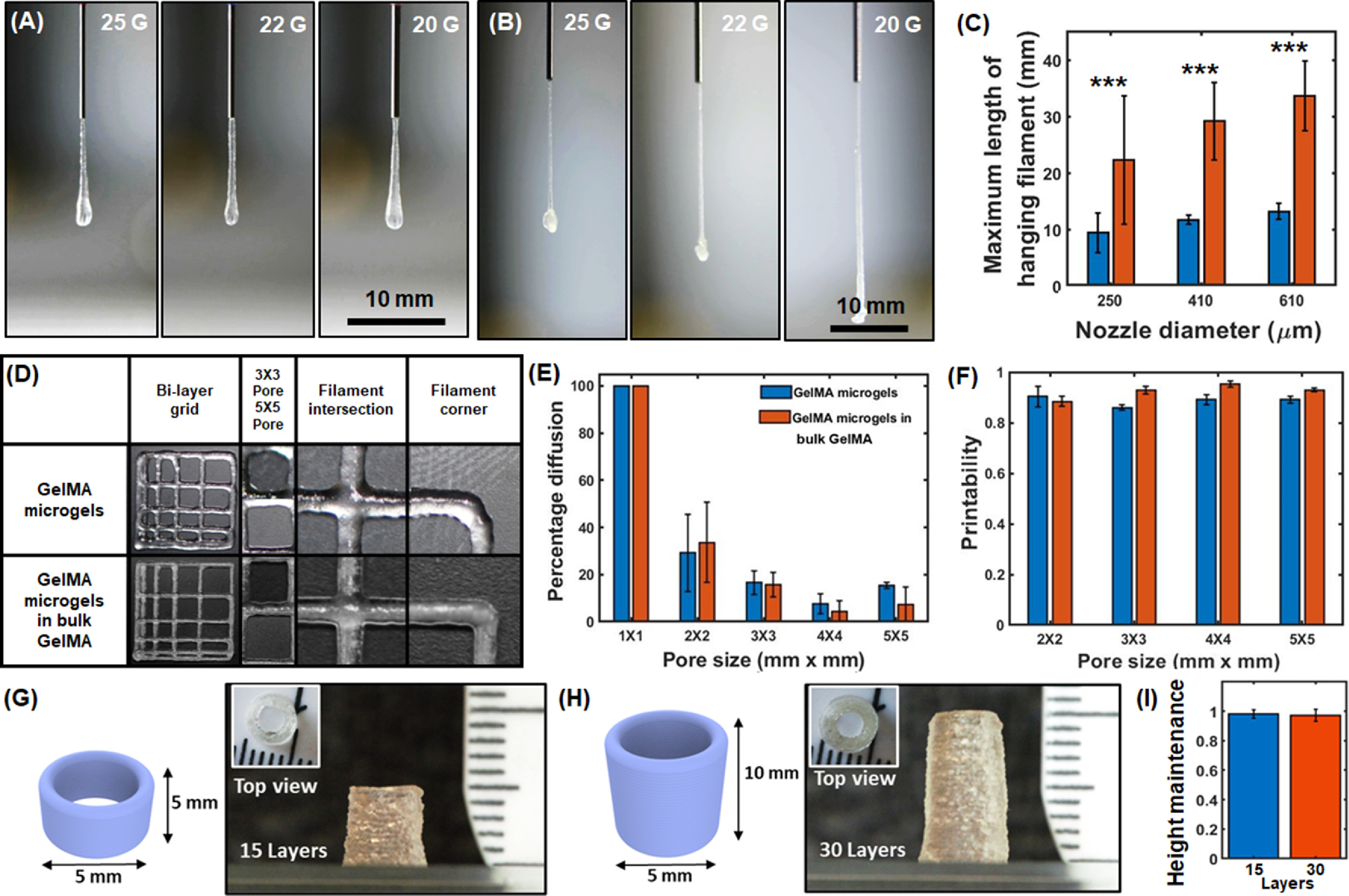
Filament length, fusion, and printability tests for multiple bioinks including GelMA microgels and GelMA microgels loaded in bulk GelMA. Representative images of hanging filaments for (A) GelMA microgels, (B) GelMA microgels loaded in a bulk GelMA hydrogel, (C) quantification of the maximum length of hanging filaments for the two bioinks. (D) 3D Bioprinted grid structures of various known dimensions. (E) The percentage diffusion of bioprinted filaments, and (F) printability of both bioinks (*n*= 3, *p* ^***^ < 0.001). 3D Bioprinted hollow cylinders with a diameter of 5 mm and a height of (G) 5 mm and (H) 10 mm, leading to varying layer numbers. (I) Height maintenance of hollow cylinders as an indicator for shape fidelity.

In the second application, microgels were used as a bioink and a support bath for extrusion-based bioprinting. Based on rheological characteristics and preliminary extrusion testing, only GelMA microgels were taken further for their assessment as a printable bioink because Alg and Alg–GelMA microgels alone were not extrudable as they showed water phase separation, did not form filaments, and caused nozzle clogging. The characterization tests for the extrusion of GelMA microgels revealed acceptable printability and shape fidelity, which was further improved after loading GelMA microgels in bulk GelMA. The hanging filament test showed that GelMA microgels had a capacity to form filaments, which is necessary for bioprinting as shown in figures 6(A) and (C). Further, the addition of bulk GelMA into GelMA microgels significantly increased (*p* ⩽ 0.001) the maximum length of hanging filaments thus increasing the robustness of the bioink for extrusion (figures 6(B) and (C)). It was also found that 22 G needles formed thinner filaments than 20 G needles while 25 G needles tended to clog. Therefore, 22 G needles were used for all characterization experiments pertaining to extrusion-based bioprinting.

The filament fusion test was then performed by 3D bioprinting grid structures of different dimensions. The results revealed that the percentage diffusion was greater than 0, which was expected as the filaments slightly deformed and diffused after deposition. Pores of 1 mm × 1 mm grid structures were completely closed resulting in a pore area of 0. Therefore, the diffusion percentage was taken as 100%. However, the percentage diffusion was less than 40% for the 2 mm × 2 mm grid structures, which decreased further to under 20% for larger pores as shown in figures 6(D) and (E). The printability of both bioinks was determined to be greater than 0.8 (figure 6(F)) indicating that they have potential to be utilized as an extrudable bioink for 3D bioprinting applications. In addition, the filament collapse test was performed, where filaments were continuously extruded or bioprinted in air. The results showed that bioprinted structures, particularly with the bioink comprising GelMA microgels loaded in bulk GelMA matrix, could maintain their shape integrity for up to 6 mm pillar distance without any external support indicating their robustness for bioprintability (figure S6). Furthermore, shape fidelity was evaluated by layer stacking of the bioink comprising GelMA microgels loaded in bulk GelMA as hollow cylinder models. Self-standing stable cylinders with a base diameter and a height of 5 mm (15 layers, figure 6(G)) and 10 mm (30 layers, figure 6(H)) were 3D bioprinted. Figure 6(I) shows the height maintenance factor (*H*_m_). The results indicated that *H_m_* was close to 1 for the 3D bioprinted constructs, indicating high shape fidelity.

**Figure 7.**
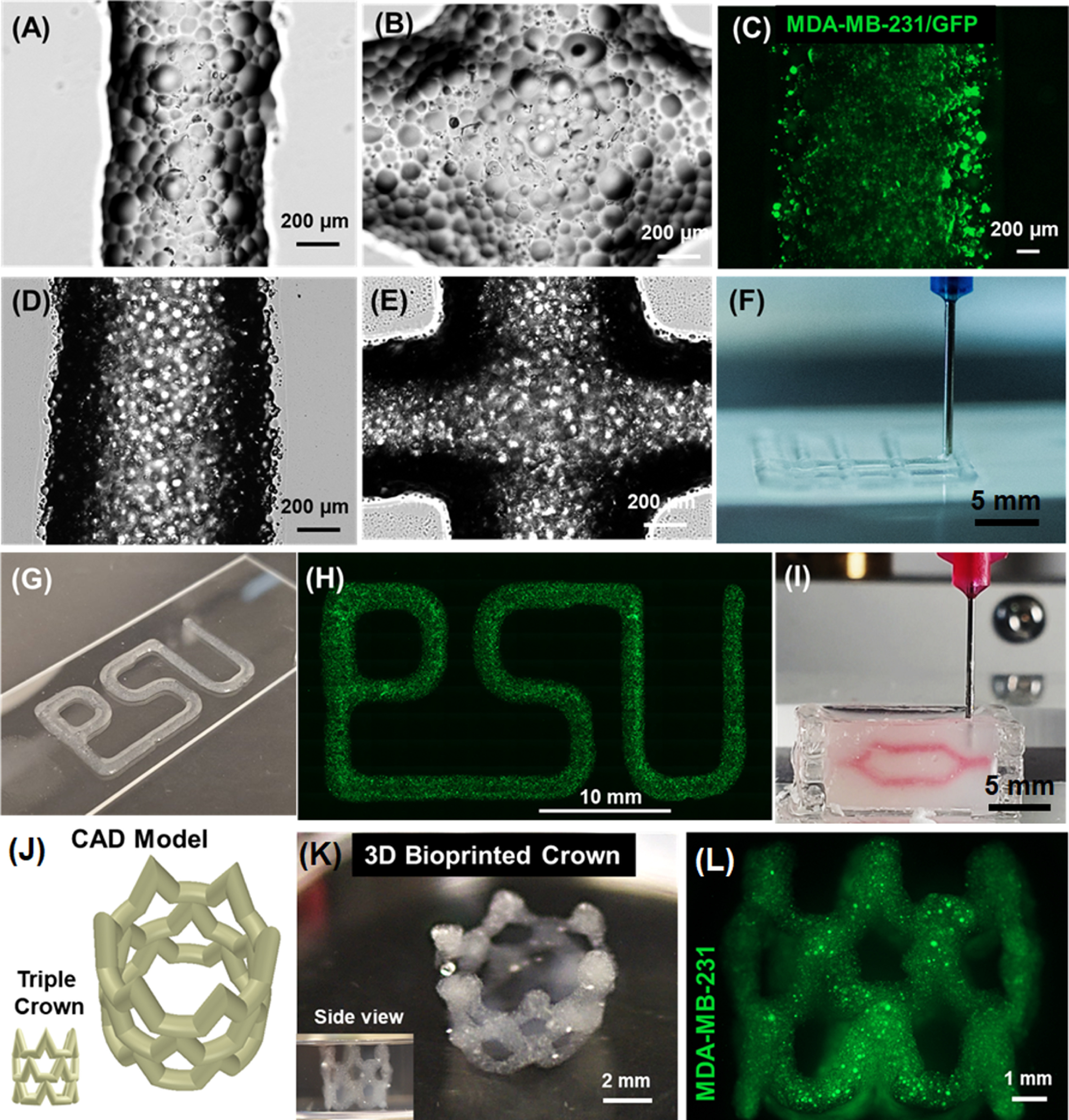
Visual demonstration of bioprinted constructs. Brightfield images showing (A) bioprinted filaments and (B) intersections made of GelMA microgels; (scale: 200 *µ*m). (C) Micrograph of GFP^+^ MDA-MB-231 cells encapsulated in bioprinted GelMA microgels (scale: 200 *µ*m). Brightfield images of (D) bioprinted filaments, and (E) intersections of GelMA microgels loaded in GelMA. (F) 3D Bioprinting of bilayer grid structures. (G) A photograph and (H) a fluorescent image illustrating cell encapsulating GelMA microgels bioprinted in the shape of ‘PSU,’ the abbreviation of Penn State University. (I) Embedded bioprinting of a branched structure inside a support bath made of Alg microgels. 3D Bioprinted complex-shaped crown constructs with their (J) 3D computer-aided design (CAD) model and (K) macroscopic photographs. (L) GFP^+^ MDA-MB-231 cells encapsulated in GelMA microgels loaded in bulk GelMA were viable after bioprinting.

After validating that GelMA microgels were bioprintable on their own (figures 7(A) and (B)) or when loaded in bulk GelMA (figures 7(D) and (E)), GFP^+^ MDA-MB-231 cells were successfully encapsulated in GelMA microgels and bioprinted as shown in figure 7(C). Similar bilayer grid structures were successfully fabricated in centimeter scale (figure 7(F)) and cell encapsulating GelMA microgels was bioprinted in the shape of the abbreviation of Penn State University ‘PSU’ (figures 7(G) and (H)), which confirms the printability and scalability of these constructs made of cell encapsulating microgels. In addition, HDFs encapsulated in GelMA microgels loaded in bulk GelMA were found to be ∼95% viable in bioprinted 3D grid structures (see figure S7 for representative LIVE/DEAD images).

Further, embedded bioprinting within Alg microgels was also successfully executed, where Xanthan gum was bioprinted into a branched network to demonstrate the potential of microgels as a support bath (figure 7(I)). Additionally, the presented approach enabled the fabrication of 3D complex-shaped constructs. MDA-MB-231 cells were encapsulated in GelMA microgels, which were then loaded in bulk GelMA, and the resulting bioink was used for 3D bioprinting of a triple-crown structure inside the Alg microgel support bath (figure 7(J)). The bioprinted crown structures showed high fidelity where the hollow structures maintained their integrity without collapse even after the removal of Alg microgels, indicating the structural stability of bioprinted constructs (figure 7(K)). Figure 7(L) shows the encapsulated GFP^+^ MDA-MB-231 cells in bioprinted structures.

**Figure 8.**
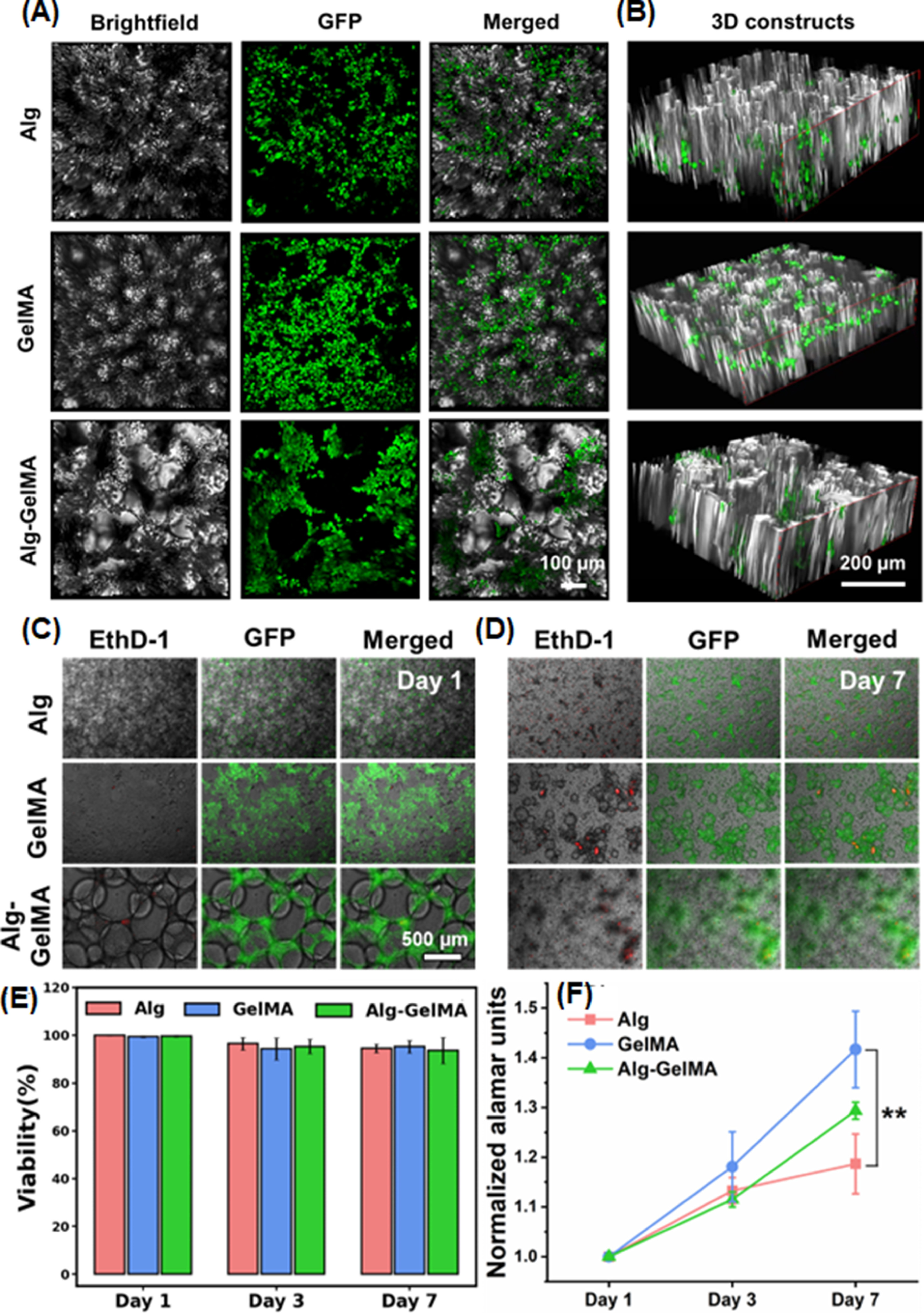
Microscopic images of GFP^+^ MDA-MB-231 cells seeded on microgels. (A) Images showing GFP^+^ MDA-MB-231 cells seeded on microgels under brightfield, fluorescence, and merged, and (B) the distribution of these cells in 3D microgel-based constructs. (C) Representative images of GFP^+^ seeded cells (green) and EthD-1-stained dead cells (red) on Days 1 and 7. (E) Quantification of viability of GFP^+^ MDA-MB-231 cells seeded on microgels at Days 1, 3, and 7. (D) AlamarBlue assay showing proliferation of GFP^+^ MDA-MB-231 cells over a week culture (*n*= 3; *p* ^**^ < 0.01).

In the last application, we used Alg, GelMA and Alg–GelMA microgels as scaffolds and seeded GFP^+^ MDA-MB-231 cells on them. We evaluate the biocompatibility of microgels by assessing the viability and proliferation of MDA-MB-231 cells. Cells seeded on microgels showed homogenous distribution after 6 h of seeding, as shown in figure 8. They could evenly surround the microgels and fill the voids between microgels in 3D (video S2). For the viability assessment, GFP^+^ cells were stained with EthD-1 to detect the dead cells as indicated by the red fluorescence signal (figures 8(C), (D) and S8). MDA-MB-231 cells exhibited viability of ∼98% on Day 1, ∼95% on Day 3, and ∼90% on Day 7 (figure 8(E)), which readily adhered to and proliferated among the microgels in interstitial void spaces. In addition, the AlamarBlue assay showed ∼1.15 and ∼1. Two-fold increase in the metabolic activity for cells on Days 3 and 7, respectively, for Alg microgels, ∼1.2 and ∼1. Four-fold increase in the metabolic activity of cells on Days 3 and 7, respectively, for GelMA microgels; and ∼1.1 and ∼1. Three-fold increase in the metabolic activity for cells on Days 3 and 7, respectively, for Alg–GelMA microgels (figure 8(F)). On Day 7, MDA-MB-231 cells seeded on GelMA microgels showed significantly higher proliferation as compared to cells seeded on Alg microgels (*p* ⩽ 0.01).

## 4. Discussions

Microgels are swollen macromolecular networks that differ significantly from typical colloids, such as micelles, vesicles, flexible macromolecules, and rigid nanoparticles. This is mainly because of the presence of a crosslinker that holds the microgel together and provides structural integrity and control over their characteristics [29]. The microgel fabrication technique described here is advantageous for cell encapsulation, bioprinting and scaffolding since it opens the possibility to develop biomaterials on a scale of tens to hundreds of microns in a high-throughput manner. It is challenging to work with bulk self-assembled polymeric gels in *in-vitro* culture or after *in-vivo* implantation without generating cracks and heterogeneities. In consequence, it would be anticipated that these heterogeneities will predominate the movement of media or physiological fluids throughout and within the materials. Here comes the role of the assembled microgels as they can modify their volume and shape in response to external stimuli (such as pressure, light, electrochemical stimulus, temperature, and pH), which enable them to reversibly tune their physicochemical characteristics.

In the current study, we successfully produced microgels of varied sizes by changing the air pressure flowing through the outer channel of the co-axial nozzle system while the polymer solution flows through the core channel. This co-axial nozzle system resulted in the formation of droplets via the mechanism of atomization. These droplets immediately get crosslinked when they reached the collector and thus formed stable microgels. The flow rate of the polymer solution was optimized to be 100 *µ*l min^−1^. In our preliminary studies, we observed the formation of large-sized microgels when the flow rate was in the range of 200–500 *µ*l min^−1^. These large microgels tended to break easily during bioprinting. On the other hand, microgels were extremely small when we used lower flow rates (10–50 *µ*l min^−1^). These microgels could not be used for cellular encapsulation. Thus, to obtain stable microgels in a suitable size range for bioink development and cell encapsulation, a constant flow rate of 100 *µ*l was used. Herein, Alg microgels were formed by flowing alginate through the core nozzle and microgels were collected in the CaCl_2_ bath. In an early study, Pravinata *et al* produced calcium alginate microgel particles (∼100 nm) using a jet homogenizer for highly turbulent mixing of two liquid streams of sodium alginate and CaCl_2_ solution [30].

For GelMA microgels, the GelMA solution containing the LAP photoinitiator flowed through the inner nozzle and droplets were collected in an oil bath, where microgels were formed due to water-in-oil emulsion and crosslinked using the 405 nm light exposure. Nichol *et al* fabricated cell-laden GelMA microgels down to 100 *µ*m resolution with fidelity and robustness by assembling microgels at the surfactant-containing oil–water interface followed by secondary crosslinking via UV light using Irgacure 2959 photoinitiator [31]. In our study, it is pertinent to note that GelMA from fish and porcine sources was used in combination. This was because we needed continuous flow of GelMA, and the porcine GelMA (typical gelling and melting points of porcine gelatin range from 20 °C to 25 °C and 28 °C to 31 °C, respectively) exhibited physical crosslinking at room temperature that makes it hard to flow while fish GelMA (typical gelling and melting points of fish gelatin range from 8 °C to 25 °C and 11 °C to 28 °C, respectively) exists as liquid at room temperature due to its lower gelling and melting temperatures but has weak crosslinking on its own [32]. Therefore, we formulated a composite hydrogel by mixing them to get the required flow properties and mechanical stability at room temperature. Importantly, we used gelatin from animal sources as they are widely used and tested even in clinical trials due to their compatibility, wide availability, and low costs as compared to human gelatin [32].

Further, Alg–GelMA microgels were produced in this study by flowing an LAP-containing blend of alginate and GelMA solution through the nozzle and collecting it in the CaCl_2_ bath and exposing to 405 nm light to accomplish dual crosslinking. To the best of our knowledge, Alg–GelMA microgels have not been reported previously in the literature.

Herein, we studied the effect of change in air pressure on the size of formed microgels. For this, different air pressure levels ranging from 40 to 180 kPa were investigated and the results indicated that at >100 kPa, the microgel size was smaller than desired, on the other hand, they were too large at ⩽60 kPa. Therefore, we preferred 100 kPa for further studies to get microgels at a consistent size, which was required for cellular encapsulation and bioprinting. In a study, Akbari *et al* developed a microfluidic multiplier droplet maker capable of producing ∼50 *µ*m microgels with a single channel throughput of up to 75 g per day for 8 *μ*m poly(ethylene glycol diacrylate) microgels at a drop generation frequency of 3.1 MHz. When the oil phase was driven through the micropillar array, shear forces from the oil phase caused the continuous stream of the cell suspension to break up into droplets [9]. Here, we used air as our continuous phase and found that increasing the air pressure resulted in decreased microgel size. Recently, Li *et al* reported a high-throughput approach, where they used a multi-channel rotating system to generate alginate microgels ranging from 100–1000 *µ*m in size [33], but the reported setup was very complex as compared to the one reported in our study.

The formed microgels were then characterized for the rate of microgel production and circularity. The results showed that ∼3800, ∼65 000, and ∼7000 microgels s^−1^ were produced for Alg, GelMA, and Alg–GelMA, respectively. For comparison with the literature, Morimoto *et al* reported mass production of calcium-alginate particles with a rate of around 171 particles s^−1^ with a size range of 100–300 *µ*m [34]. Additionally, the circularity assessment in our study indicated that the formed microgels had a wide range of circularity. GelMA microgels showed the highest circularity, closely followed by Alg microgels. Alg–GelMA microgels showed the least circularity, which might be due to the improper blending of the two polymers and their incomplete crosslinking, especially in the core of these large microgels. Therefore, the main factors determining the final size of microgels include the polymer flow rate, air pressure at the co-axial nozzle, and microgel composition.

Further, a systematic rheological analysis was carried out to examine the three types of microgels and evaluate their applicability as an extrudable bioink for bioprinting or as a support bath for embedded bioprinting. The decrease in the viscosity of GelMA microgels upon increasing the shear stress signifies that the applied stress overcame the inter-microgel interactions of GelMA. It led microgels to move relative to each other, and after crossing the yield point, microgels were no longer elastic and behaved like a viscous fluid. Further, the amplitude sweep test also validated that GelMA microgels behaved as a yield stress gel, and their mechanical regime transformed from elastic to viscous nature near 7% strain. This shear thinning effect is crucial for uniform extrusion through a nozzle during bioprinting [35]. Furthermore, an equally important requirement is the recovery of microgels to their original arrangement on the build plate after exposure to a higher stress inside the nozzle. This is called self-healing capability and is needed for bioprinted construct to remain intact. GelMA microgels demonstrated self-healing capability highlighting the fact that they rearranged and packed themselves again immediately after the removal of higher shear rates. Alg microgels also showed shear thinning behavior as their viscosity decreased with increasing shear stress. Since we utilized Alg microgels as a support bath for bioprinting, this shear thinning functionality was crucial as it allowed smooth movement of the needle inside the bath without breaking it. Further, the self-healing capability of these microgels resulted in the quick recovery into their original shape after the removal of higher shear stress. Therefore, Alg microgels were successfully used as an appropriate support bath for embedded bioprinting. Like GelMA and Alg microgels, Alg–GelMA microgels also exhibited shear thinning and self-healing properties. Therefore, they have potential to be used as an extrudable bioink as well as a support bath.

Although rheological data showed promising results for microgels as a yield stress bioink and a support bath, bioprinting experiments should be performed to verify its applicability in bioprinting. During bioprinting, there can be additional factors like gravity, surface tension, clogging, and drying of bioink, which play a crucial role in extrusion and can alter the final extrusion outcomes [36, 37]. During our study, it was noticed that GelMA microgels were suitable for bioprinting only up to a few layers and therefore not recommended for developing hanging constructs. However, when GelMA microgels were loaded in bulk GelMA, the composite bioink gained the ability to facilitate 3D self-standing constructs as validated with the significantly longer filaments and ability to form stable hollow cylinders of up to 10 mm in height (30 layers) shown in figure 6. Alg microgels showed their potential as a support bath owing to their promising yield stress properties [25, 26]. Herein, we also demonstrated the scalability, self-standing ability, and shape fidelity of the 3D bioprinted complex-shaped crown constructs using cell encapsulated GelMA microgels loaded in bulk GelMA and bioprinted into the Alg support bath, which was removed later indicating the feasibility of the proposed strategy.

Microgels added benefits in terms of extrusion and filament formation as compared to bulk hydrogels. This is mainly due to the microgel properties such as higher porosity, interparticle interaction, surface-to-volume ratio, and the rate of response to stimuli. Additionally, cells may experience lower shear stress when they are encapsulated in microgels as compared to when they are in bulk gel. Increasing the porosity of microgels improves the rate of molecular diffusion, convectional fluid flow of nutrients and soluble signaling mediators, and allow better cell infiltration. Furthermore, filament formation was challenging when using only microgels due to the space among microgels. They were prone to coagulation due to inter-particle interactions, which coagulated the nozzle as well as yielded non-uniform filaments. Towards improving the printability, several packing methods have been explored including microgel jamming or centrifugation at higher speeds [38]. However, this limits their application for cell encapsulation. On the other hand, microgels loaded in a bulk hydrogel formed more stable filaments as the hydrogel occupied the space among microgels thus improving their extrudability without impairing the viability of encapsulated cells.

For the scaffolding application, GFP^+^ MDA-MB-231 cells were seeded on microgels, which showed immediate penetration after their topical seeding on microgels based on the acquired 3D *z*-stacked images. Cell penetration into microgels was dictated by the pore size distribution, interconnectivity, and packing. Herein, no separate jamming of microgels was performed except for the centrifugation during the processing and washing of microgels. This contributes towards a facile and rapid approach of microgel production. The cellular viability for all microgels was found to be ∼90–98 over a period of seven days indicating the biocompatibility of all three microgel types. However, cell proliferation with GelMA microgels was shown to be significantly higher as compared Alg. This is plausibly due to the inherent material properties of polymers. Alg is negatively charged, and as cell surface is also negatively charged, it limits the ability of cells to bind, and this has been explored for alginate’s application in immune-isolation of pancreatic islets [39, 40], among other applications. GelMA, on the other hand, is a biocompatible material with tunable physical characteristics. It has native extracellular matrix like integrin-binding motifs and matrix metalloproteinase sensitive groups, which make it suitable for cell attachment and proliferation [41]. Moreover, its polymerization using LAP allows crosslinking in visible or near UV range (405 nm) making it safer for cells. The limitations of Alg in terms of bioactivity could be overcome by combining it with GelMA. The concentration and crosslinking methods of the two different hydrogel-precursor solutions can be tuned to enable a higher resolution of cell-laden microgels to recreate the ideal microenvironment, cell spreading, and organization. The formed Alg–GelMA microgels supported higher cell proliferation as compared to Alg alone.

The presented microgel fabrication technique is advantageous for cell encapsulation, 3D bioprinting, and scaffolding applications since it helps define microgels on a scale of tens to hundreds of microns. Encapsulating cells in microgels allows their easy transfer from one container to another without inducing major stress on them. These microgels could potentially encapsulate different cell types or use various microgel types encapsulating different cell types forming multilayered structures. The application of the produced microgels in the current study could be extended to encapsulate single cells. Current cell encapsulation methods typically produce high polymer-to-cell ratios and lack control over the hydrogel’s mechanical properties [42]. Mao *et al* reported a microfluidic-based method for encapsulating single cells in a ∼6 *µ*m layer of alginate that increases the proportion of cell-containing microgels by a factor of ten, with encapsulation efficiencies over 90% [43].

Based on the obtained results, we hypothesized that the size of microgels influences their physicochemical properties including colloidal stability, circularity, rheology, cell encapsulation efficiency, and printability thus impacting their overall performance and functionality. In our study, we found that the stability of microgels followed the order Alg–GelMA < Alg < GelMA (figure S4). Correlating the stability with microgel size, it was observed that larger microgels (>500 *µ*m) arbitrarily encapsulated smaller microgels thus making their reproducibility difficult. Whereas smaller microgels (<100 *µ*m) were usually not suitable for encapsulation applications because of physical restraints. However, owing to their smaller size and low yield-stress, these microgels found their applications as support bath for embedded bioprinting. From our experiments, we observed that an optimum range (50–250 *µ*m) favors stable colloidal suspension for our targeted applications including cell encapsulation and bioprinting. Therefore, GelMA microgels (78% in the 50–250 *µ*m range) showed suitable viscosity, circularity, cell encapsulation, yield point, and self-healing for bioprinting as compared to smaller Alg microgels (55% in the 0–50 *µ*m and 45% in the 50–250 *µ*m range) and larger Alg–GelMA microgels (13% in the 50–250 *µ*m and 62% in the >500 *µ*m range) (figure S9). In addition, the printability of GelMA microgels were found to be satisfactory, which was improved upon when microgels were loaded in bulk GelMA (figure 6). Overall, microgel size is a pertinent factor to be considered for specific applications. However, apart from size, other factors including surface charge, interparticle interaction, degree of crosslinking, and porosity also play a crucial role in determining the stability and functionality of microgels [29].

The presented biofabrication strategy provides a facile and rapid approach without any complex or expensive consumables and accessories for scalable/high-throughput production of spherical microgels, with or without cell encapsulation, that can find a wide range of applications, some of which have been demonstrated here. There is a clear tradeoff between the microgel size and production efficiency. As per the aim of our study, we utilized a platform for easy, faster, and cost-effective method of microgel production. A heterogenous population of microgels with a wide size range were produced that might find applications, where size homogeneity of microgels is not crucial. Nevertheless, the size of microgels could be tuned by optimizing parameters including nozzle size, polymer flow rate, pressure, and crosslinking strategies, to produce uniform-sized microgels. Depending on the application, the size could be tuned or methods such as filtering out microgels of selective size could be opted. Controlling the size could also be extended towards achieving single cell encapsulated microgels. In the case of Alg–GelMA microgels, their size and shape could not be tuned enough as stable microgels were not formed at pressures above 80–100 kPa and the successfully formed microgels at 60 kPa degraded in PBS within five days. Thus, its application might be limited to strategies involving rapid delivery using larger microgels. Towards improved usability of microgels, their cell encapsulation efficiency and the number of cells per microgel ratio could be further controlled by tuning the cell concentration used in the precursor solution and the size of microgels. However, the size of microgels is linked to the air pressure and this has an upper cap as cells might disintegrate at higher air pressure levels.

## 5. Conclusion

In this study, Alg, GelMA and Alg–GelMA microgels were produced using an ACAD in a continuous and high-throughput manner and their utilities were exemplified in multiple applications. Herein, we showed a much higher rate of microgel production particularly for GelMA microgels, where microgel size depended on the air pressure. The results indicate that the proposed Alg microgels have the potential to be used as a prospective support bath material while GelMA microgels have potential for direct extrusion both on their own or loaded with bulk GelMA. Microgels supported cellular attachment, viability, and proliferation, particularly GelMA microgels, and showed high encapsulation efficiency with the capability to encapsulate single to multiple cells. Overall, this study illustrates a facile, affordable, and rapid method for microgel production, which can be tuned further for various potential applications including encapsulation and delivery of cells and to fabricate cell-laden structures via 3D bioprinting.

## Data Availability

All data that support the findings of this study are included within the article (and any supplementary files).
